# Measuring Relevant Information in Health Social Network Conversations and Clinical Diagnosis Cases

**DOI:** 10.3390/ijerph15122787

**Published:** 2018-12-09

**Authors:** Albert Moreira, Raul Alonso-Calvo, Alberto Muñoz, José Crespo

**Affiliations:** 1 Biomedical Informatics Group, Departamento de Lenguajes Sistemas Informáticos e Ingeniería de Software & Departamento de Inteligencia Artificial, Escuela Técnica Superior de Ingenieros Informáticos, Universidad Politécnica de Madrid, 28660 Madrid, Spain; albertmoreira@infomed.dia.fi.upm.es; 2 Faculty of Medicine, Universidad Complutense de Madrid, 28040 Madrid, Spain; albertom@pdi.ucm.es

**Keywords:** social media, health, patients, healthcare professionals, collaboration measurement, medical terminologies, conversation participation indicators

## Abstract

The Internet and social media is an enormous source of information. Health social networks and online collaborative environments enable users to create shared content that afterwards can be discussed. The aim of this paper is to present a novel methodology designed for quantifying relevant information provided by different participants in clinical online discussions. The main goal of the methodology is to facilitate the comparison of participant interactions in clinical conversations. A set of key indicators for different aspects of clinical conversations and specific clinical contributions within a discussion have been defined. Particularly, three new indicators have been proposed to make use of biomedical knowledge extraction based on standard terminologies and ontologies. These indicators allow measuring the relevance of information of each participant of the clinical conversation. Proposed indicators have been applied to one discussion extracted from PatientsLikeMe, as well as to two real clinical cases from the Sanar collaborative discussion system. Results obtained from indicators in the tested cases have been compared with clinical expert opinions to check indicators validity. The methodology has been successfully used for describing participant interactions in real clinical cases belonging to a collaborative clinical case discussion tool and from a conversation from a health social network. This work can be applied to assess collaborative diagnoses, discussions among patients, and the participation of students in clinical case discussions. It permits moderators and educators to obtain a quantitatively measure of the contribution of each participant.

## 1. Introduction

The area of health information systems is constantly growing. Besides the private information systems that are maintained by hospitals and health institutions organizations, in recent years the proliferation of health social networks or online health communities have proven its worth both for patients themselves and for research, i.e., for crowdsource health research studies [1]. However, the trustworthiness of sources is very important in health environment. Clinical processes—including diagnosis—are often based on articulation and effective communication among physicians of various disciplines across time and space. Several factors may lead to a lack of understanding of what constitutes effective communication, thus, leading to medical errors that threaten patient safety. Specifically, in developing countries [2,3], some diseases often cannot be treated in time because of the lack of a basic health infrastructure. The lack of adequate support for diagnosis can lead to incorrect administration of medications or serious complications and, in some cases, may lead to the death of the patient.

Despite their importance, collaborative computer-based discussions of clinical cases are still underused in clinical environment. On the other hand, patients are actively using health networks available [4] that empower patients in terms of: emotional support, information about their disease, social comparison, or emotional expression (e.g., the PatientsLikeMe social network [5] that, when patients share their experience with other patients, has reported benefits in patient outcomes).

While in professional settings the discussion of clinical cases was traditionally performed on-site, contemporary clinical work is often distributed among multiple stakeholders across different places and at different times. Progress in communications and technologies enables this interaction to be carried out remotely using computers or mobile phones; however, effective communication in medical settings remains a major challenge. In situations when the context is more complex and requires greater agility for diagnosis and treatment, collaboration [6,7,8] has been an option to encourage the exchange of experiences in order to increase the chances of positive results [9].

The focus of this paper is to present a methodology for quantifying the interactions in clinical diagnose conversations. This methodology defines a set of indicators supported by biomedical vocabularies and natural language processing (NLP) techniques [10], as well as traditional collaborative environments measurements [7]. Being able to measure the impact of interactions of different participants of discussion would help to discriminate who is helping in building relevant medical knowledge. A significant aspect of this methodology is to translate user participation within clinical conversations to a set of numerical values corresponding to different aspects of the conversation. Obtaining a numerical representation facilitates the comparison of the interactions of participants. This way, it can be evaluated whether a collaborative interaction has been truly collaborative or, on the other hand, just one participant has carried out practically all the conversation. This characterization can be used: to assess collaborative diagnoses among professionals and discussions among patients by moderators of health social networks, and to evaluate how medical students participate in clinical case discussions in an educational environment. To the best of our knowledge, there are no similar methodologies that have been proposed in the literature.

## 2. Materials and Methods

The present work evaluates the possibility of quantifying the interaction of each participant in clinical conversations, measuring each individual’s contributions and the evolution of group work. In network discussions, mechanisms that support cooperative diagnoses are also considered [11,12]. To successfully evaluate collaborative conversations, several aspects from different areas must be taken into account [13,14]. 

### 2.1. Indicators for Measuring Contributions in Clinical Diagnosis Cases

The goal of this work is to obtain a numerical representation of the value of each contribution in a clinical conversation. Relevant amount of valuable information from the words written in a conversation has to be extracted and represented numerically. 

Besides using some classical indicators from collaborative environments, like the number of words in a contribution or effort needed by each participant in their messages construction, we have defined additional indicators that measure contextual information about contributions in a conversation that could be found in some scenarios or tools. These additional indicators want to measure previous research done by a participant before writing a message, i.e., if the user has inspected multimedia or images loaded in the discussion; or, if she has studied any research paper related to the case. We have also defined new indicators strongly related to the specific area of discussions: health. Thus, in addition to common collaborative aspects, specific biomedical knowledge appearing in discussions should be measured. Natural language processing (NLP) tools for extracting medical knowledge based in standard terminologies are used for obtaining the amount of clinical information provided in a contribution, taking then into account the number of concepts appearing in contributions.

Indicators that we have defined to quantify the interaction in clinical conversations are: user participation, object manipulation, interaction variation, and previous research. Additionally, one indicator and two ratios for measuring participants’ activity have been defined: diagnostic relevance indicator, new concepts by contribution ratio, and diagnostic correctness ratio. Each of these indicators and ratios are explained in the following subsections.

#### 2.1.1. User Participation Indicator

The participation of users in textual communication or implementations on an artefact can be collected without variations derived from the environment or the domain [15,16]. Written text, for example, is a way of getting information from an environment. This is an important factor for knowing the level of performance of the individual in the construction of an artifact, in this case, the diagnosis or solution of the conversation.

The level of participation of the user is measured until the moment he or she stops talking or is interrupted by another person, as if it were a shift system. A shift begins when a person starts talking alone. In summary, the most appropriate way to measure is by the number of messages; that is, each message sent can be considered one episode of speaking. Counting the number of words can also be an option to find the amount of participation, since the words are separated by spaces. Thus:

Participation quota = number of words or messages sent(1)

Group conversation is the sum of all individual participations. If the goal is to obtain the amount of each participant, an average can be calculated as

Group participation = Σ Amount of participation of each user(2)

Individual participation rate = Participation quota/Group participation(3)

In the context of our work, this aspect is reflected directly through the comments on each separated case. Obviously, the influence of the user in case A would not lead to a greater participation in case B.

#### 2.1.2. Object Manipulation Indicator

The amount of work performed by one user within an online conversation application is by itself a good indicator of her interest in the consecution of the task [17]. Similarly to the participation indicator mentioned above, this measure allows us to establish whether users are following the case and in what manner. We define the manipulation of objects by

Amount of manipulation = frequency of objects touched by a user,(4)

Amount of manipulation of the group = Σ Amount of manipulation,(5)

In practical terms with a discussion tool, this manipulation of the objects is given by the use of the attached media and links in the cases up for discussion.

In a wider domain, it would also be possible to measure the level of user interaction with other elements of the collaborative application that do not refer exclusively to clinical cases, i.e., changes in user profile, documentation, and searches in cases.

#### 2.1.3. Interaction Variation Indicator

Another factor that defines the richness of one discussion is the variation of the interaction [18]. A user can actively participate in a specific discussion even if he or she has never discussed other cases.

Interaction variation = Number of commented cases/Number of cases,(6)

This can occur for a variety of reasons, such as the number of cases related to patient’s illness or the professional’s specialty. On the other hand, it may be a warning about the quality of the remaining cases. If they are more complete, they can intimidate other users to participate.

A derived measurement of this aspect would be to calculate the user’s participation during a certain period. This could be an indicator of the level of application fidelity.

#### 2.1.4. Previous Research Indicator

Adequate preliminary research can accelerate and promote efficiency in clinical case discussions. When this indicator is not used, it can discourage participants and compromise the quality of the final result. The preliminary investigation process can benefit both the author of a case and the participants in the interaction.

In the context of the methodology, the following indicator quantifies the number of cases that the user and the group access in order to calculate the manipulation of clinical cases in the public database repository

Amount of previous research = Frequency of case manipulation of the repository by a user,(7)

Amount of previous group research = Σ Amount of previous research,(8)

As discussed, the interaction occurs through the action of consulting previous cases of the repository.

The amount of related cases investigated by participants can be difficult to measure and it is limited to the work inside the health social network or collaborative platform used. Thus, for the implementation of this indicator, the inclusion of a contextualization method is recommended. Contextualization would provide links to public repositories such as PubMed, which provides free access to access to MEDLINE [19,20], as well as magazines and e-books. MEDLINE is the National Library of Medicine (NLM) journal citation database. It includes more than 26 million citations and abstracts from the biomedical literature. Contextualization of clinical cases implies using natural language processing techniques on the case title and description in order to extract medical concepts that appear and finally provide publication links containing those concepts [10,20]. The implementation of this indicator could be even more complete, i.e., measuring the time spent by one participant in each previous case through the supervision of the web session. This action aims to increase the degree of certainty about the participant’s work in the reading of each previous related case.

If no contextualization methods are available in collaborative environment, the indicator would need an activity report filled by the participant containing citations of research performed supporting his contribution.

#### 2.1.5. Diagnostic Relevance Indicator

Current indicators can measure the interaction of a user within a collaborative decision. The usefulness of the suggestions or interventions of participants, however, is usually not only due to the number of words or interventions performed. For this reason, we have defined a new specific indicator within the collaborative scope among health professionals: the diagnostic relevance indicator.

Through the use of automatic annotations of the contributions and diagnostic proposals of the participants using natural language processing techniques, we can quantify the clinical contributions (diagnoses and symptoms) that appear in an interaction that have not appeared previously in the case

(9) $$\begin{array}{ll} \mathrm{Amount}\;\mathrm{of}\;\mathrm{new}\;\mathrm{clinical}\;\mathrm{information}= & \mathrm{number}\;\mathrm{of}\;\mathrm{standard}\;\mathrm{clinical}\;\mathrm{concepts}\;\mathrm{of}\;\mathrm{contribution}\\ & \mathrm{did}\;\mathrm{not}\;\mathrm{previously}\;\mathrm{exist}\;\mathrm{in}\;\mathrm{the}\;\mathrm{case}, \end{array}$$

The moderation of clinical cases requires experience related to the specialty being discussed. In cases where the action zone is still undefined, or when managing a multidisciplinary case, this aspect is even more critical, as it is possible for there to be more than one moderator support.

Each contribution to a case should be annotated using standard vocabularies and the concepts that have not appeared before in the case, either in questions or affirmations, should also be associated with each contribution. A possible variation would be to divide these contributions by the type of clinical concept used, whether it is a diagnosis (or suggestion thereof), or rather concerns diagnostic results or symptoms. As a complement, the indicator could culminate in a ranking.

#### 2.1.6. New Concepts by Contribution Ratio

Based on the indicators explained above, derived indicators can be obtained, such as the percentage of new diagnostics by contribution to the total of an intervention.

(10) $$Newconceptscontributionratio=\frac{Amountofnewclinicalinformation}{Userparticipationindicator},$$

In the context of the presented methodology, this factor reflects the diagnostic suggestions or contributions. This ratio could help to characterize the participants, i.e., if they are brief in their messages but provide information, or on the contrary, if the texts of their contributions are very broad but do not add new clinical suggestions or information.

#### 2.1.7. Diagnostic Correctness Ratio

Because cases, as discussed in the previous subsections, must be concluded by a moderator, the participant who suggested the final diagnosis in the case can be marked.

(11) $$\mathrm{Diagnostic}\;\mathrm{correctness}\;\mathrm{ratio}=\frac{\mathrm{correct}\;\mathrm{diagnostic}\;\mathrm{contributions}}{\mathrm{number}\;\mathrm{of}\;\mathrm{cases}\;\mathrm{in}\;\mathrm{which}\;\mathrm{it}\;\mathrm{has}\;\mathrm{contributed}},$$

This ratio, similarly to the previous one, could be used to characterize the participants of the discussions by creating a rank of expertise in the discussion application. Table 1 summarizes indicators and ratios that have been defined in the methodology.

### 2.2. Application of Indicators for Measuring Relevant Information in Clinical Diagnosis Cases and Conversations

**Figure 1:**
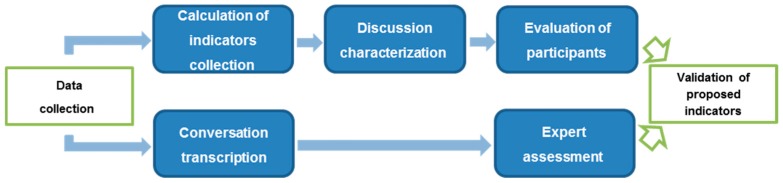
Workflow of application of the methodology (upper path) and expert validation (lower path).

Once the characteristics to be measured have been concretized into a set of indicators, it is necessary to define how these indicators must be used in clinical discussions for different phases of the interaction evaluation. Those different stages are depicted in Figure 1.

#### 2.2.1. Data Collection

The initial step in order to measure the interaction of participants in a collaborative diagnosis is to obtain the data for calculating certain indicators. Theoretically, the data collection could be applied during a face-to-face discussion using recordings and a supervisor that could be manually gathering the defined indicators. Defined indicators could also be applied partially to ‘a posteriori’ discussion, or in those cases when there is no possibility of collecting all defined indicators, by using only the texts of the interactions.

#### 2.2.2. Calculation of Indicators

To validate the relevance of the defined indicators, experiments were performed considering two discussion tools. The expected result is a similar pattern for both.

In the construction of the methodology, two forms of calculation have been tested. Even if they are compensated with standard deviations and variances, they result in a measurement difficulty. To increase the assertion of the measurements in the context of a discussion, we used coefficients using two types of comparisons. In the first comparison, the basis is the sum of the measurement values commonly used for group measurements. For example, the variation indicator of group interaction can use this basis for the coefficient. In the second comparison, the base used corresponds to the highest value, normally used for individual measurements. For example, the individual object manipulation indicator can use this basis for the coefficient.

#### 2.2.3. Characterization of Discussion

The proposed methodology can be applied to both synchronous and asynchronous discussions, although the latter format improves the greater inflow of new participants and contributes to a more lasting and effective interaction.

To contribute to the automation of the process, the discussion occurs practically without the intervention of the moderator figure. In specific situations of experimentation, in which the discussion may range far from the expected objective, the moderator may arbitrate, e.g., by suggesting that the reasoning and the discussion be directed in a different way. Although concern has been mentioned, the aim of the experiment is to encourage discussion naturally without intervention. Ultimately, the discussion functions similarly to a forum; however, there are a number of mechanisms available to evaluate the particularities inherent in the interaction between the participants in a more precise way.

#### 2.2.4. Evaluation of Participants and Discussion

The evaluation of a participant is obtained from the measurement indicators and should be presented based on the results of each coefficient. To quantitatively characterize the conversational style of participants in a discussion, tables of indicators are used by ordering the participants’ values for each indicator. The conversational style is evaluated using three aspects:

(i) Participatory style, based on indicators: (i.1) user participation (using the individual participation rate in the case), (i.2) object manipulation, and (i.3) interaction variation.
(ii) The interest of a participant in review literature about the case, as measured by the previous research indicator.
(iii) Clinical contribution, which estimates participant interactions specifically related to clinical knowledge, using the diagnostic relevance indicator.

#### 2.2.5. Conversation Transcription

The group of messages that compose the discussion are translated and printed so that the expert could read, and interpret them in paper format. In addition to the messages, the transcription contains all available information about manipulated images, cases accessed, and investigations accessed by participants in the conversation.

#### 2.2.6. Expert Assessment

To evaluate the numerical results obtained using the methodology indicators, a clinical expert (external to the cases) was asked to describe the participation of the members involved in the case. Due to the difficulty of finding experts available and willing to contribute, it was decided to carry out the assessment with one expert but with a broad experience in illnesses treated in the cases. The expert is a general medicine clinical doctor, 58 years old, and active in clinical practice.

Particularly, the expert has considered the work dynamics during the interactions, without presenting him indicators collected from conversation in the cases. To define the work dynamics, the expert provided information regarding: user participation, the value and impact of the contributions of each user, and the relevance of his or her previous research (access of objects such as images and literature review). 

The expert reviews the transcription of the conversation (Section 2.2.5), and freely makes comments about the interventions of participants. Afterwards, the assessment of the expert is compared with the evaluation of participants (Section 2.2.4) for validation, as displayed in Figure 1.

### 2.3. Platforms Used in the Experiments

To test the validity of the proposed methodology two clinical online platforms have been selected: Sanar [21], and PatientsLikeMe. 

Sanar is a collaborative diagnosis web tool, where each member of the group can use a web browser to create and comment on health cases. It was designed to support the building of medical knowledge among health professionals in a global context. The main idea is to support health professionals in sharing knowledge about clinical cases, resulting in more informed opinions about the case and, consequently, allowing a more consistent diagnosis. 

The Sanar platform was selected primarily because it could be modified to implement all the proposed indicators. Experiments using Sanar were the basis for analyzing the relevance of the indicators proposed in the methodology, with a focus on arriving at a common diagnosis in a real environment among clinical professionals. The experiments are also important to identify what helps and what hinders the development of a discussion, which can serve as a basis for reinforcing the relevance of each indicator for a more efficient interaction. 

PatientsLikeMe is a well-known health social platform designed specifically for interactions among patients with a common disease. In this system, patients report their health information, which is presented as a coherent graphic sample in their profile. Member profiles are posted where other members can access them, providing a basis for the exchange of passive information and active dialogue among patients. Internal implementation and databases of PatientsLikeMe were not available; thus, only some of the proposed indicators can be applied.

## 3. Results

The presented methodology is the result of the learning accumulated from interventions of participants. Three examples are presented. The first and second use the collaborative diagnose tool Sanar [21]; and the third example uses a clinical discussion taken from the PatientsLikeMe platform.

The two studies carried out with Sanar were based on the indicators defined for the methodology, concretely, the calculations of all indicators were implemented in Sanar system. For these two selected conversations, a group of five volunteers participated in the execution of both experiments. All the participants were general medicine clinical doctors, with ages in a range from 25 to 38 years old and located in different geographical areas in Brazil.

The third test, from PatientsLikeMe, also intends to use the proposed methodology for already completed cases to which some of the defined indicators cannot be applied, as mentioned before.

### 3.1. Quantification of the First Test Case Using Sanar

This first clinical case is a discussion from Sanar system, entitled “rash, joint pain, and conjunctivitis in a 25-year-old male patient”. The clinician that opened the case included patient’s images. Results of the experiment conducted indicate an appropriate use by members, more details are shown below.

#### 3.1.1. Calculation of Indicators

• User Participation Indicator—Amount and time of each turn of discussion in a case

**Figure 2:**
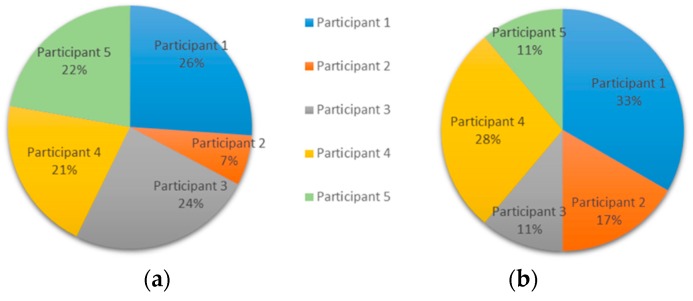
Experiment 1—User Participation Indicator: individual participation rate (**a**) in words, and (**b**) in messages.

**Figure 3:**
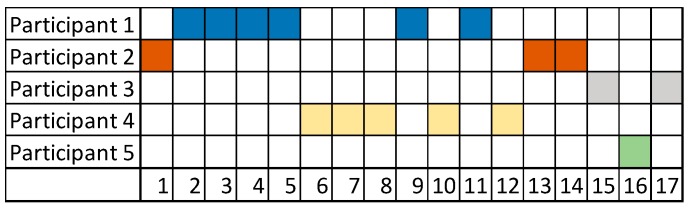
Experiment 1—User Participation Indicator: distribution of messages.

The experiment resulted in an exchange of 18 messages over a day. The commitment of the users, as indicated by the frequent exchange of messages in a short time, was noted. The participation of each user is depicted in Figure 2a based on the number of words, and Figure 2b based on the number of messages during the conversation. Additionally, Figure 3 shows the order of messages of the conversation.

• Object Manipulation Indicator—Sum of the amount of manipulation of objects

In general, access to objects occurs uniformly and often at least twice in 80% of cases.

As shown in Table 2, the users seem to be careful to access the objects related to the case before joining the discussion. In some situations, the user accesses each object inherent to the case more than once. This may have been a way to deepen knowledge, as considered in the research criteria.

In two situations, the user accesses the same image several times. In the first scenario, the observation has generated evidence that this action was an attempt to understand the purpose of the image, possibly unsuccessfully. The same pattern was seen in the case of the second participant, followed by a relevant question in the area of interaction. This may be an indication that the user has reported that the image generated a dubious interpretation to the user or that it was poorly captured.

As seen, the attention to detail in the pursuit of knowledge is a characteristic that is often inherent in a health professional.

• Interaction Variation Indicator—Amount of participation in various medical cases

In this indicator, Table 3, shows the number of cases where each participant have interacted within Sanar.

• Previous Research Indicator—Number of cases manipulated

Specifically, in relation to this indicator, the access to previous reports indicates the interest of the participants in this functionality. The increase in the level of the discussion was an aspect perceived by the author and by the participants themselves, as a result of the survey carried out as the second part of the first experiment. The results obtained from this indicator open possibilities for the selection of cases in order to prioritize the discussion of those considered more complex or controversial. Results are shown in Table 4.

• Diagnostic Relevance Indicator—Clinical concepts mentioned in the case discussion

During the evaluation of the case, texts of the discussion were processed using MetaMap tool (developed by the U.S. National Library of Medicine (NLM)) to automatically extract relevant clinical concepts, as it can be observed in Table 5. Then first concept found in discussion was Dengue, followed by the Zika virus concept. It is noteworthy that the Yellow Fever disease was also cited.

#### 3.1.2. Evaluation of Participants

Different interpretations of the contribution of the participants can be established from the calculated indicators. We will discuss participation, research and clinical contribution aspects based on the numerical results of the indicators:

(i) Participatory style. Participants 1, 4, and 5 presented a similar participatory style behavior. The individual participation rate shown in Figure 1 indicates that Participants 1, 2, 3, and 5 have a similar word-based participation. However, Participants 1 and 4 have greater message-based participation than the rest. On the other hand, Participant 2 shows low contribution in the conversation, which may indicate low interest or initiative. It is noteworthy that the interventions of Participant 3 appeared only at the end of the discussion, when the diagnosis was almost defined.

The object manipulation indicator shown in Table 2 indicates that Participant 1 had accessed objects (images) several times. Participants 4 and 5 also accessed several objects, but Participants 2 and 3 had low interaction with those attached objects.

The interaction variation indicator in Table 3 shows that Participants 1, 4, and 5 were more active in past discussions in the Sanar system than Participants 2 and 3.

(ii) Previous research indicator displays in Table 4 that all participants navigated to PubMed for reading several suggested papers, especially Participants 1 and 4.
(iii) The clinical contribution of Participant 1 is the highest. He or she, together with Participant 4, provided most of the clinical terms appearing in the conversation. Participants 3 and 5 also used some clinical concepts, although they only introduced one new concept each.

As summary, considering the 18 generated messages, there was a dialogue between two participants (1 and 4) as if they were directly discussing. They provided the first occurrence of most of the clinical concepts, especially Participant 1. The comments of Participants 2 and 4 were based on observations previously made by other participants, and they used concepts already introduced. Participant 2 had low input in the case. Participant 1 was also the most active in reviewing research of related cases and in inspecting the complementary data provided in the case.

#### 3.1.3. Expert Assessment

The expert interprets user interventions using the transcription of the conversation. Regarding the participation criteria, the expert asked about the age of each user. He then noted that younger users tended to risk more in contributions. Even at this point, it was concluded that Participant 1 had a greater participation in the experiment. The expert considered that the number of messages exchanged in the case was sufficient. Also in this context, when observing the activities log, the expert mentioned that Participants 2 and 3 either did not realize that images were available for visualization or were not interested. Regarding the contribution criteria, the specialist said that all participation is important for diagnostic detection, but User 1 plays a key role in establishing the main option for diagnosis and influencing others about his point of view, especially the undecided ones. On the previous research criterion, the expert preliminarily infers that Participant 1 had undoubtedly made a preliminary inquiry. When the participant realizes that he or she was right, he or she reinforces that the previous investigation is valid to increase the possibilities of contributing significantly to the discussion.

Thus, in general terms, expert’s comments are similar to those outlined from indicators in the previous General Evaluation section. Since indicators and ratios were not presented to the expert, this confirms the idea of that expert’s line of reasoning is aligned to the way that the indicators and ratios were constructed in the methodology. The presence of a specialist was important to confirm the relevance of the indicators and ratios proposed, as they are similar to the reasoning presented in the evaluation made by the expert.

### 3.2. Quantification of the Second Test Case Using Sanar

The second clinical case is also a discussion from Sanar system, entitled “57-year old patient with diplopia”. The clinician that opened the case included symptoms’ images. Results of the experiment conducted indicate an appropriate use by members, more details are shown below.

#### 3.2.1. Calculation of Indicators

• User Participation Indicator—Amount and time of each turn of discussion in a case

**Figure 4:**
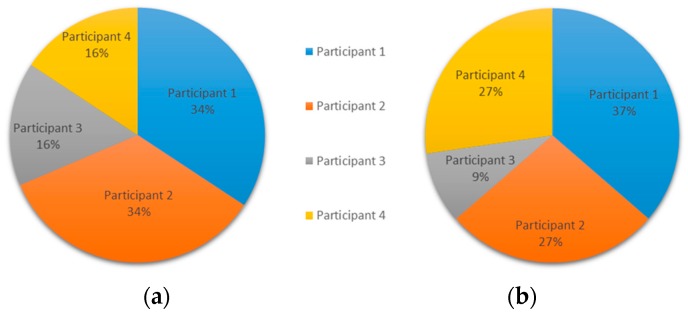
Experiment 2—User Participation Indicator: individual participation rate (**a**) in words, and (**b**) in messages.

**Figure 5:**
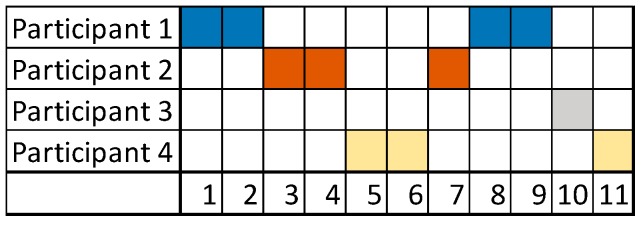
Experiment 2—User Participation Indicator: distribution of messages.

The experiment resulted in an exchange of 11 messages over a day. With the information from Experiment 1, it is possible to say that the participation indicator was slightly lower, even if it was sufficient for the detection of the diagnosis. The participation during experiment 2 is shown in Figure 4a based on the number of words, and Figure 4b based on the number of messages. Additionally, Figure 5 shows the distribution of messages ordered by time.

• Object Manipulation Indicator—Sum of the amount of manipulation of objects

Objects related to the case before submitting the opinions were accessed but with lower intensity compared to case 1, as shown in Table 6.

In addition to the descriptions, there was at least 50% access to the images in the interactions. In some situations, the user accesses each object inherent to the case more than once. This situation occurred in order to deepen knowledge, following the research criteria.

• Interaction Variation Indicator—Amount of participation in various medical cases

As in the first experiment, although there was interaction with one case, one participant accessed to read and comment on another different published case. The interaction variation indicator can be observed in Table 7.

• Previous Research Indicator—Number of cases consulted

The information in Table 8 confirms the interest of the participants contextualization functionality. As in the previous experiment, the level of the discussion was increased. This indicator tends to generate better results with the participant who stands out in the discussion. Their greater participation in the subject under discussion motivates the deepening in previous research and other cases.

• Previous Research Indicator—Number of cases consulted

As can be observed in Table 9, the first concept that was commented on was circulatory disorders, followed by congenital strabismus. In addition to these, cerebrospinal fluid, angiography, ultrasound, and electromyography (EMG) were also been mentioned.

#### 3.2.2. Evaluation of Participants

As in the first experiment, the results can establish different interpretations of the contribution style of the participants. We will discuss participation, research, and clinical contribution aspects based on the numerical results of the indicators:

(i) Participatory style. Participants 1 and 2 presented a similar participatory style behavior. The individual participation rate shown in Figure 1 depicts that Participant 1 is the most active one, whereas Participant 3 had low participation in conversation.

The object manipulation indicator exposed in Table 6 indicates that Participants 1 and 4 had accessed all objects attached to the case.

The interaction variation indicator presented in Table 7 shows that all participants had previously participated in a similar number of cases in the Sanar system.

(ii) The previous research indicator appearing in Table 8 displays that Participant 1 was the most active one in reviewing related scientific research papers.
(iii) Clinical contribution of Participants 1 and 4 were high. They also provided most initial occurrences of the clinical terms appearing in the conversation. Participants 3 and 5 also used some clinical concepts, although they only introduced one new concept each.

Considering the 11 messages of this conversation, Participants 1 and 2 were the most participative ones, but it can be remarked that Participant 1 had a more curious style in research activities. Additionally, the clinical contribution indicator shown in Table 9 specifies that Participant 2 did not provide any novel clinical concept in the discussion. Participants 1 and 4 introduced most of the clinical concepts that conducted to the final diagnosis. Participant 3 presented low values in participation and research measures, although his or her opinion was important to arrive to the final diagnosis of diplopia. 

#### 3.2.3. Expert Assessment

As in Experiment 1, an expert was invited to evaluate the discussion of this case, taking into account criteria such as participation, contributions, and previous research. The method used for evaluation was also the same: the group of messages that compose the discussion was transcribed and attached to the general register with information about manipulated images, cases accessed from the tool and investigations from the previous research repository. As in the previous test case, the expert did not have access to the indicators proposed in the methodology.

Regarding the participation criteria, the expert said that, in general, this case had little participation, but that would be worth evaluating it because the final diagnosis was correct. He said that in less complex cases it is common for there to be fewer messages. He also stated that interaction between Participants 1 and 2 occurs throughout the discussion, but that Participant 4 makes important contributions to address the reasoning for the diagnosis. Observing the record table with data on object manipulation, regarding images, the expert noted that the low rate of manipulation of images by Participant 2 combined with the large amount of participation in the messages can be a risk (in the sense that Participant 2 can influence users about a diagnosis without being sure). Regarding the contribution criteria, the expert said that the discussion was brief because the diagnosis of this case was simpler and was practically defined in the first messages. Regarding the previous research criterion, the expert had the opportunity to examine the data access to repository cases and commented that previous research had a major impact on preparing the user in this discussion.

Again, the specialist’s analysis is important to confirm the relevance of the proposed indicators and coefficients.

### 3.3. Quantification of the Test Case Using PatientsLikeMe

The third experiment uses a conversation extracted from PatientsLikeMe health network with the title “Blood pressure quick short-term fix”. This case aims to demonstrate that it was possible to proceed with the application of the methodology in other discussions systems than Sanar, as detailed below.

#### 3.3.1. Calculation of Indicators

• User Participation Indicator—Amount and time of each turn of discussion in a case

This indicator was obtained from a manual message count. The experiment resulted in an exchange of nine messages over eight days. In comparison with other cases of PatientsLikeMe, this is higher than average.

• Object Manipulation Indicator—Number of manipulated objects

While PatientsLikeMe does not present this information, thus, this indicator did not generate data for the evaluation. Regardless, an important point is that the solution allows the publication of images, although this did not occur in the evaluated case.

• Object Manipulation Indicator—Sum of the amount of manipulation of object

Since PatientsLikeMe does not present this information, similarly to previous indicator, this indicator did not generate data for the evaluation. As mentioned in the previous point, no images were published in the evaluated case.

• Interaction Variation Indicator—Amount of participation in various medical cases

This study considers the interaction with one case. The user cannot see the number of messages posted per user profile as in other forums.

• Previous Research Indicator—Number of cases manipulated

Previous queries in the discussion indicate the interest of the participants in previous research. The increase in the level of the discussion is a perceived factor from an analysis of the participation of users over time. As mentioned, the results obtained from this indicator open possibilities for the selection of cases in order to prioritize the discussion of those considered more complex or controversial. However, at the moment of writing this work, PatientsLikeMe does not present a contextualization tool and indicator cannot be calculated.

• Diagnostic Relevance Indicator—Clinical concepts mentioned in the case discussion

During the discussion period, the main focus of the dialogue was on the treatment for the reported problem. In this case, beyond the initial advice given by a user, other recommendations were made as described in Table 10.

#### 3.3.2. Evaluation of Participants

**Figure 6:**
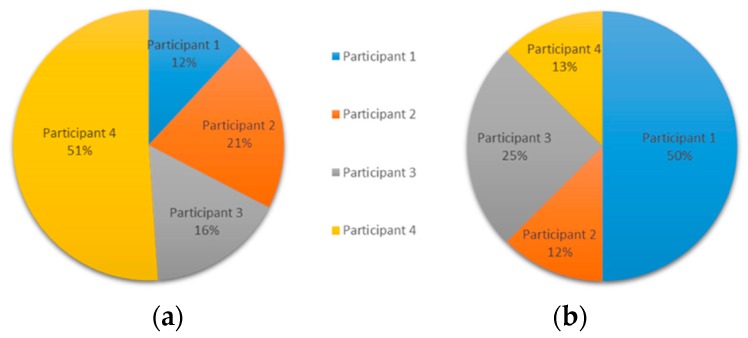
Experiment 3—User Participation Indicator: individual participation rate (**a**) in words, and (**b**) in messages.

The analysis of the experiment allowed both objective and subjective visions, opening possibilities for more assertive future conclusions. Using the indicators proposed in the methodology, we discuss the interactions of the participants in terms of the numerical results obtained:

(i) Participatory style. In this case, Participant 1 had the highest participation in terms of messages, while Participant 4 showed the highest value of word-based participation, as can be observed in Figure 6.

Both the object manipulation indicator and the interaction variation indicator could not be calculated for this experiment (since we have not access to internal implementation of PatientsLikeMe, as mentioned in Section 2.3).

(ii) The previous research indicator could not be obtained in this experiment (same reasons as above).
(iii) The clinical contributions are shown in Table 10. This table indicates that Participant 2 did not suggest any new clinical concept. On the other hand, the other three participants provided one new clinical concept each.

**Figure 7:**
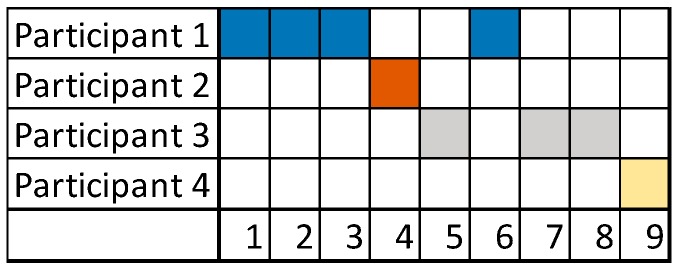
Experiment 3—User Participation Indicator: distribution of messages.

Considering the nine messages, although the conversation is not long, we can conclude that Participant 1 was trying to guide the conversation by sending most of the messages. Later, Participant 3 contributed with one brief message with a clinical suggestion. Finally, Participant 4 stated the final diagnosis in his contribution of only one message. The distribution of messages, depicted in Figure 7, is important in this case to evaluate the contributions of different participants. Figure 6 depicts participation of users in experiment 3, Figure 6a based on the number of words, and Figure 6b based on the number of messages. Figure 7 shows the distribution of messages of different users ordered by time.

#### 3.3.3. Evaluation of Participants

To finalize the evaluation, the same process was performed in the PatientsLikeMe experiment. The second experiment tested the criteria of participation, contributions and previous research. The same method as in Experiment 1 was used for evaluations.

As in Case 2, the expert considered that there was low participation, although he found a more distributed participation among all contributors. He has detected that the level of discussion was sufficient for the situation, although the profile of the group would not be of health professionals but probably formed by patients. He commented that Participant 1 started and normally stood out in the discussion, although in this case this position is shared with Participant 3. Regarding the log with data about object manipulation, the expert asked if there would be something with a similar effect in PatientsLikeMe. Regardless, he considered that the low complexity of the case and the description were sufficient elements. Concerning the criteria of contributions, the specialist identified the equal distribution of the messages over eight days. He said that the contribution of Participant 4 was important because it strengthened the final diagnosis. Regarding the previous research criterion, the expert commented that more experienced participants are likely to have gained knowledge from previous discussions in order to contribute with more assertive opinions.

In this experiment, the specialist’s analysis was important because in a conceptual way, it has a reasoning that is similar to what has been proposed in the indicators and ratios. This is seen above all in the previous research criterion.

## 4. Discussion and Conclusions

A novel methodology has been proposed in order to facilitate the characterization of discussions and participants in collaborative diagnosis tools and also to enable its application to health social networks. To the best of our knowledge, there are no similar methodologies that have been proposed in the literature. This methodology aims to model interactions performed by several participants in a conversation, particularly in the health domain. Conversations can be quantitatively characterized using the proposed indicators in an objective manner, and for example, it can be identified whether a collaborative interaction has been really collaborative. The methodology can be applied to assess collaborative diagnoses among professionals, health related discussions among patients, and the way medical students contribute in discussions of clinical cases. Therefore, evaluators (in educational environments) and moderators (in health social networks) could obtain an objective measure of the work of each participant of a health-related discussion. The benefits of the application of this methodology could be for both medical students and patients. While clinicians would reinforce their diagnose skills during training, patients will benefit from more accurate advice in health social networks.

The experiments presented in the Section 3 indicated the difficulties in the synchronization of the team and its contribution to an autonomous process. Motivating health professionals is a constant challenge, in part due to their irregular work schedules.

To achieve an effective communication, indicators were generated in order to quantify the interaction and establish a ranking that evaluates the participation during the interaction. These indicators were successfully applied for describing participant interactions in real clinical cases belonging to a collaborative clinical case discussion tool. Additionally, a case extracted from the PatientsLikeMe health social network was evaluated with respect to some of the proposed indicators, obtaining satisfactory results on describing participant’s interaction and relevance. 

It was observed that the indicator denominated ‘previous research’ should be an important component for discussion aimed at the diagnosis of clinical cases. Thus, future work can further investigate this indicator to evaluate which cases generate the greatest impact on the discussion.

## Figures and Tables

**Table 1:** Summary of indicators related to the collaborative aspect that the indicator is related to within the methodology.

Indicator	Contribution	Meaning
User participation	Individual	Comments sent and participation in discussion turns
	Group	Amount and time of each turn of discussion in a case
Object manipulation	Individual	Amount of manipulation of objects
	Group	Sum of the amount of manipulation of objects
Interaction variation	Individual	Amount of participation in various medical cases
Previous research	Individual	Preliminary investigation for knowledge improvement
Diagnostic relevance	Individual	New clinical concepts mentioned in the discussion
New concepts contribution ratio	Individual	Relevance of contribution made
Diagnostic correctness ratio	Individual	Assertiveness of contributions made

**Table 2:** Experiment 1—Object Manipulation Indicator.

Participant	Objects Manipulated Once (5 Points)	Total	Objects Manipulated More Than Once (1 Point Each Time)	Total	Objects Not Manipulated (−5 Points)	Total	TOTAL
Participant 1	3	15	2	2	1	−5	12
Participant 2	1	5	2	2	3	−15	−8
Participant 3	1	5	1	1	3	−15	−9
Participant 4	2	10	1	1	2	−10	1
Participant 5	2	10	1	1	2	−10	1

**Table 3:** Experiment 1—Interaction Variation Indicator.

Participant	Cases Involved
Participant 1	4
Participant 2	1
Participant 3	1
Participant 4	2
Participant 5	2

**Table 4:** Experiment 1—Previous Research Indicator.

Participant	Cases Manipulated in the Repository
Participant 1	6
Participant 2	1
Participant 3	2
Participant 4	4
Participant 5	3

**Table 5:** Experiment 1—Diagnostic Relevance Indicator.

Participant	Clinical Concepts Provided
Participant 1	3
Participant 2	0
Participant 3	1
Participant 4	2
Participant 5	1

**Table 6:** Experiment 2—Object Manipulation Indicator.

Participant	Objects Manipulated Once (5 Points)	Total	Objects Manipulated More Than Once (1 Point Each Time)	Total	Objects Not Manipulated (−5 Points)	Total	TOTAL
Participant 1	2	10	1	1	0	0	11
Participant 2	1	5	0	0	1	−5	0
Participant 3	1	5	1	1	1	−5	1
Participant 4	2	10	0	0	0	0	10

**Table 7:** Experiment 2—Interaction Variation Indicator.

Participant	Cases Involved
Participant 1	2
Participant 2	1
Participant 3	1
Participant 4	1

**Table 8:** Experiment 2—Previous Research Indicator.

Participant	Cases Manipulated in the Repository
Participant 1	5
Participant 2	2
Participant 3	1
Participant 4	2

**Table 9:** Experiment 2—Diagnostic Relevance Indicator.

Participant	Clinical Concepts Provided
Participant 1	2
Participant 2	0
Participant 3	1
Participant 4	2

**Table 10:** Experiment 3—Diagnostic Relevance Indicator.

Participant	Clinical Concepts Provided
Participant 1	1
Participant 2	0
Participant 3	1
Participant 4	1
